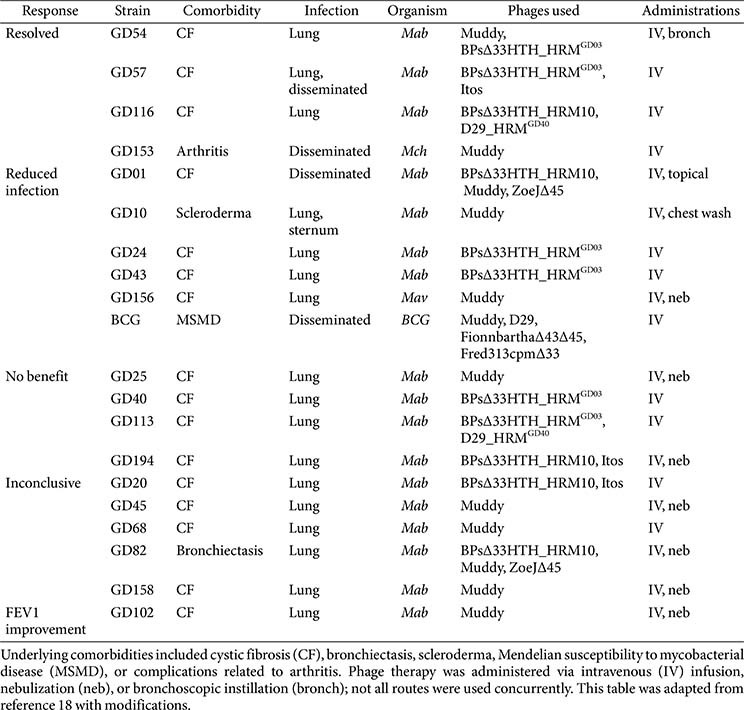# Clinical and Translational Perspectives on Bacteriophage Therapy for Nontuberculous Mycobacterial Diseases

**DOI:** 10.4014/jmb.2509.09030

**Published:** 2025-12-29

**Authors:** Anwesha Ash, Cheol Moon, Jichan Jang

**Affiliations:** 1 Division of Life Science, Department of Bio & Medical Big Data (BK21 Four Program), Research Institute of Life Science, Gyeongsang National University, Jinju 52828, Republic of Korea; 2 Department of Clinical Laboratory Science, Semyung University, Jecheon 27136, Republic of Korea

**Keywords:** Bacteriophage therapy, *Mycobacterium abscessus*, antibiotic resistance, compassionate use, phage–antibiotic synergy, nontuberculous mycobacteria

## Abstract

*Mycobacterium abscessus* complex (MABC) infections are among the most intractable challenges in clinical mycobacteriology because of their extensive intrinsic and acquired antibiotic resistance. Recent studies on compassionate use and a systematic 20-patient cohort study demonstrated that bacteriophage therapy is safe and generally well-tolerated, and it has been proven capable of inducing clinically meaningful improvements. Nevertheless, patient outcomes remain heterogeneous, largely because of antibody-mediated neutralization during intravenous administration and morphotype-dependent susceptibility, with smooth variants exhibiting resistance to currently available phages. Notably, phage resistance has rarely been observed in treated isolates, suggesting that durable efficacy is achievable when guided by pretreatment susceptibility screening. Emerging strategies, including phage engineering, lytic enzyme application, and liposomal encapsulation, are being developed to overcome intracellular barriers and immune clearance, whereas phage–antibiotic combinations have displayed synergistic activity. POSTSTAMP, the first prospective clinical trial, is establishing a structured framework for standardized evaluation. Collectively, these findings suggest that current compassionate use cases and small-scale cohorts provide a foundation for integrating bacteriophage therapy as a complementary strategy alongside antibiotics in future MABC treatment regimens.

## Introduction

Bacteriophages (phage)—the viruses that infect bacteria—constitute the most abundant biological entities on earth [1]. Mycobacteriophages exhibit exceptional genetic diversity and infect a wide spectrum of *Mycobacterium* species, spanning slowly growing pathogens such as *Mycobacterium tuberculosis* to rapidly growing organisms like *Mycobacterium abscessus*. Since the first isolation of phages active against *M. tuberculosis* (*e.g.*, phage D29) in early studies and the subsequent development of large‐scale mycobacteriophage discovery programs using *Mycobacterium smegmatis* mc²155 as a host, thousands of mycobacteriophages have been discovered and characterized (over 2 000 sequenced to date) [2, 3]. Genomic comparisons reveal more than 30 distinct clusters and hundreds of unique sequence types; most mycobacteriophages possess double-stranded DNA genomes and exhibit siphoviral, and more rarely myoviral, morphotypes, all of which fall within the class Caudoviricetes. These tailed bacteriophages display long, flexible, non-contractile tails that mediate host recognition and attachment, and their genomes exhibit strongly mosaic architectures shaped by extensive horizontal gene exchange and ongoing evolutionary diversification [4-7]. More recently, targeted isolation of phages infecting *M. abscessus* clinical strains has accelerated, generating libraries and engineered constructs tailored for therapeutic applications against non‐tuberculous mycobacterial (NTM) infections [2, 8].

Infections caused by *M. abscessus* complex (MABC) are increasingly recognized as formidable clinical challenges because of the bacterium’s intrinsic and acquired resistance to multiple antibiotic classes, leading to poor treatment outcomes and limited therapeutic options [9, 10]. MABC comprises three subspecies, *M. abscessus* subsp. *abscessus*, *M. abscessus* subsp. *massiliense*, and *M. abscessus* subsp. *bolletii*, which differ markedly in their antimicrobial susceptibility profiles, largely because of genetic variation in *erm(41)* encoding a ribosomal methyltransferase responsible for inducible macrolide resistance, a critical determinant of treatment response [11, 12]. Although *M. abscessus* subsp. *massiliense* typically carries a truncated, nonfunctional *erm(41)* allele and remains macrolide-susceptible, *M. abscessus* subsp. *abscessus* and *M. abscessus* subsp. *bolletii* often retain a functional allele, leading to resistance to clarithromycin and azithromycin, key components of standard nontuberculous mycobacterium (NTM) regimens [11-13].

Beyond macrolides, MABC exhibits broad intrinsic resistance to aminoglycosides, β-lactams, rifamycins, and tetracyclines. This innate resistance is mediated through multiple mechanisms, including enzymatic drug modification (*e.g.*, aminoglycoside-modifying enzymes, β-lactamases), reduced envelope permeability, and active drug efflux [14]. In addition to intrinsic resistance, MABC can further acquire resistance during treatment through de novo mutations or horizontal gene transfer. Clinically relevant examples include mutations in *rrl* and *rrs*, conferring high-level resistance to macrolides and aminoglycosides, respectively, and overexpression of efflux systems following prolonged drug exposure [14]. The convergence of intrinsic and acquired resistance severely undermines the efficacy of current multidrug regimens, which often include parenteral agents, such as amikacin, imipenem, and tigecycline in combination with oral macrolides [15]. Importantly, treatment success rates remain unacceptably low, particularly for *M. abscessus* subsp. *abscessus*, and long-term antibiotic therapy is frequently complicated by toxicity and relapse [10, 16].

Given this therapeutic impasse, interest has increasingly shifted toward pathogen-specific, non-antibiotic alternatives. Among these, bacteriophage therapy has gained attention as a promising approach for managing multidrug-resistant mycobacterial infections. Phages possess several advantages over conventional antibiotics, including high specificity for their bacterial hosts, ability to replicate directly at the site of infection, and capacity to exert bactericidal activity with minimal disruption to the commensal microbiota [17, 18]. Moreover, because phages cannot infect or affect human cells, they represent a uniquely selective modality for eliminating bacterial pathogens [19, 20]. Advances in phage isolation, characterization, and genetic engineering have enabled the development of mycobacteriophage libraries and modified constructs tailored to overcome phage resistance or broaden host range [21].

In this review, we highlight key pre-clinical findings and compassionate use case reports that collectively inform the clinical utility and translational potential of phage-based strategies for MABC infection management.

## First Clinical Application of Engineered Phages in Disseminated *M. abscessus* Infection

The first successful clinical application of bacteriophages against disseminated *M. abscessus* infection represented a pivotal milestone in translating phage therapy into clinical medicine [19, 22, 23]. This case originated from the extensive mycobacteriophage repository established by Graham F. Hatfull’s group through the SEA-PHAGES program, which systematically collected and characterized more than 10,000 phages across over 150 institutions [20]. This archive became indispensable when clinicians at Great Ormond Street Hospital (GOSH) sought therapeutic options for two cystic fibrosis patients who had undergone double-lung transplantation and subsequently developed disseminated *M. abscessus* infections [24].

Among the two patients, only one-a 15-year-old girl infected with *M. abscessus* subsp. *massiliense*-ultimately received phage therapy. The infecting strain, designated GD01, was isolated from her clinical specimens and found to be resistant to nearly all available antibiotics [19, 24]. In the initial phase, screening of a representative subset of archived phages identified Muddy, a naturally lytic phage originally isolated from an environmental sample, as the only phage capable of efficiently lysing GD01 [19, 24]. Muddy thus represents the sole example of a non-engineered, naturally lytic phage with confirmed activity against this clinical isolate. However, its narrow host range limited its utility against other *M. abscessus* strains [19, 23, 24].

**Fig. 1:**
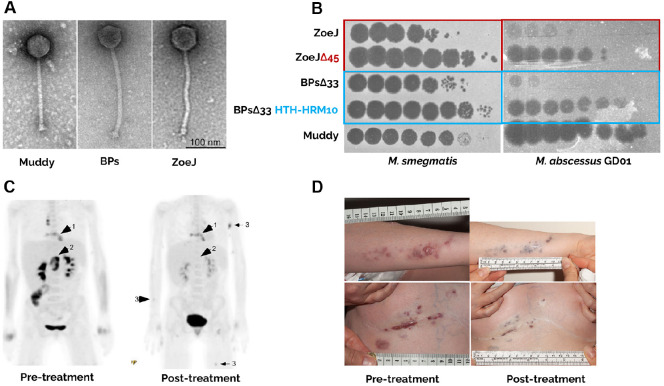
Personalized selection, engineering, and clinical implementation of therapeutic mycobacteriophages for disseminated *M. abscessus* infection. A 15-year-old girl with cystic fibrosis developed a disseminated infection caused by macrolide-resistant *M. abscessus* subsp. *massiliense*, prompting compassionate-use phage therapy. (**A**) Transmission electron micrographs of siphoviral phages Muddy, BPs, and ZoeJ, originally isolated from the Hatfull laboratory’s SEAPHAGES collection. These phages were selected as therapeutic candidates based on genomic safety, lytic activity, and modifiability (scale bar, 100 nm). (**B**) Host-range screening on *M. smegmatis* and the patient isolate *M. abscessus* GD01 showing that only Muddy and two engineered derivatives-the repressor-deleted ZoeJΔ45 and a spontaneous host-range mutant BPsΔ33HTH-HRM10 derived from an integrase-deleted background-productively infect GD01. (**C**) PET-CT imaging before and six weeks after intravenous phage administration demonstrates substantial reduction in inflammatory lesions involving sternal, hepatic, and cutaneous sites. (**D**) Clinical photographs show marked improvement of sternal and skin lesions after six months of twice-daily intravenous phage therapy (10^9^ PFU per phage per dose). The three-phage cocktail was well tolerated and resulted in significant microbiological, radiologic, and clinical improvement. Figure adapted from Dedrick *et al*. [19].

Recognizing this limitation, the Hatfull laboratory transitioned toward engineered phages to broaden therapeutic applicability. To overcome the temperate nature and host restriction of most mycobacteriophages, the team employed targeted genome modification strategies to generate obligately lytic and genetically stable variants suitable for therapeutic use. Two temperate mycobacteriophages, ZoeJ and BPs, were genetically modified by deleting their respective repressor genes (Δ45 and Δ33HTH) to generate obligately lytic derivatives (Fig. 1A) [11]. These deletions were constructed using the BRED (Bacteriophage Recombineering of Electroporated DNA) method, which allows precise markerless genome editing through homologous recombination [19]. In addition, a spontaneous host-range mutant of the engineered BPs phage (BPsΔ33HTH-HRM10) was isolated through forward genetic screening, which successfully infected GD01 through adaptive mutations in the portal gene (Fig. 1B). Subsequent genomic analysis revealed that HRM10 carried a single-nucleotide substitution (C2083T) within the portal gene, resulting in an R66W amino acid change that expanded its host range toward GD01 [19]. The final cocktail consisted of three phages-Muddy, ZoeJΔ45, and BPsΔ33HTH-HRM10-mixed in equal proportions (1 : 1 : 1), each at 1 × 10^9^ PFU per dose (total 3 × 10^9^ PFU). The cocktail was administered intravenously every 12 h for 32 weeks and applied topically to skin lesions as an adjunct. The therapy was well tolerated with no adverse effects. Within weeks, the patient showed substantial improvement, including regression of skin lesions, closure of the sternal wound, normalization of liver function, weight gain, and culture conversion of sputum and blood samples (Fig. 1C and 1D). [19, 22, 23]. No phage-resistant mutants emerged during or after treatment. Although weak antibody responses to the engineered phages were detected, the patient’s serum did not neutralize phage activity, and Muddy remained poorly immunogenic. This limited humoral response is likely attributable to the patient’s immunosuppressive therapy following lung transplantation, which reduced the capacity to mount robust antibody responses. In addition, phages are inherently less immunogenic due to their protein composition and rapid clearance from circulation, and Muddy in particular appears structurally less prone to elicit strong immune activation[19, 23, 24]. The development of engineered mycobacteriophages such as ZoeJΔ45 and BPsΔ33HTH marked a pivotal advance in establishing phage therapy as a clinically viable strategy. Genome engineering was crucial not only to convert temperate phages into obligately lytic and therapeutically reliable agents but also to ensure genomic safety through comprehensive intragenomic analyses confirming the absence of lysogenic or virulence-associated genes [19, 23, 24]. This compassionate-use case demonstrated that both natural and engineered bacteriophages can safely and effectively treat multidrug-resistant *M. abscessus* infections in humans, challenging prior assumptions that intracellular mycobacteria are inaccessible to phages and laying the groundwork for personalized, genome-engineered phage therapeutics. Importantly, since this milestone, mycobacteriophage genome engineering has advanced further with the CRISPY-BRED system, which combines BRED recombineering with CRISPR-Cas9-mediated counter-selection to achieve higher precision and efficiency in phage genome modification [25].

## Subsequent Clinical Cases Highlighting Immunological Barriers and Therapeutic Strategies in Mycobacteriophage Therapy

### Limitations of Phage Therapy: Antibody-mediated Neutralization as a Therapeutic Barrier

**Fig. 2:**
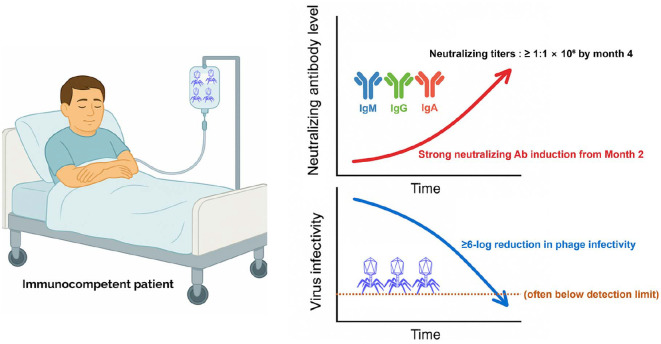
Neutralizing antibody–associated suppression of phage infectivity during intravenous therapy. Progressive amplification of the patient’s humoral response during therapy is visualized by the rise in class-switched antibodies (IgM, IgG, and IgA) and the corresponding decline in circulating phage activity. The temporal trajectories highlight the dynamic interplay between antibody induction and loss of phage infectivity, illustrating how host immunity increasingly restricts phage availability despite ongoing intravenous administration. This dataset-integrated schematic emphasizes the immunological barrier that emerges in immunocompetent individuals receiving repeated phage infusions, complementing the quantitative findings presented in the main text. This illustration was created with an AI-based illustration tool.

Although the aforementioned 2019 landmark case demonstrated the potential of engineered mycobacteriophages to treat disseminated *M. abscessus* infections, subsequent experiences have revealed important limitations [26]. The second reported case of mycobacteriophage therapy involved an immunocompetent 81-year-old man with non-CF bronchiectasis and pulmonary infection caused by macrolide-resistant *M. abscessus* subsp. *massiliense*. The infecting strain, designated GD82, exhibited a rough colony morphology, and it was closely related to the GD01 strain. Similarly to GD01, GD82 displayed broad-spectrum drug resistance, including macrolide resistance, which severely restricted therapeutic options. To identify suitable phage candidates, the isolate was screened against a large mycobacteriophage library, yielding several phages with robust lytic activity. The most active phages were Muddy and engineered derivatives of BPs (BPsΔ33HTH_HRM10) and ZoeJ (ZoeJΔ45), which were the same phages employed in the GD01 case. Guided by this susceptibility profile, a three-phage cocktail, with each phage delivered at approximately 1 × 10^9^ PFUs twice daily over a 6-month period, was intravenously administered alongside concurrent multidrug antibiotic therapy. Intravenous administration was selected because cavitary lung abnormalities were believed to limit the penetration of aerosolized phages. This rationale aligns with subsequent clinical experiences reported by the Hatfull group, where similar concerns regarding cavitary lesions informed the initial decision to employ intravenous delivery prior to transitioning to aerosolized administration [27]. Treatment produced a transient decline in sputum bacterial counts at 1 month, accompanied by modest symptomatic improvement, but efficacy subsequently waned. From the second month onward, the patient mounted strong IgM, IgG, and IgA responses against all three phages, with neutralizing titers exceeding 1:1 × 10^6^ by month 4, effectively reducing phage infectivity *in vitro* by ≥6 logs (often below the detection limit)(Fig. 2). Despite this immune pressure, bacterial isolates retained susceptibility to Muddy and the BPs derivative and displayed only intermittent resistance to ZoeJΔ, indicating that immune neutralization rather than phage resistance explained the rebound of the bacterial burden. Importantly, serial laboratory monitoring and imaging uncovered no treatment-related toxicity, and hematologic and biochemical parameters remained stable, although cavitary lesions in the right upper lobe progressed. In contrast to the earlier GD01 case, in which an immunosuppressed adolescent failed to mount neutralizing responses despite prolonged intravenous exposure, this outcome illustrates that robust humoral immunity in immunocompetent hosts represent a dominant barrier to sustained efficacy and underscores the importance of considering both the immune status and route of delivery in phage therapy [26, 27].

### Nebulized Phage Therapy for Refractory *M. abscessus* Pulmonary Disease: Clinical Outcomes and Immunological Challenges

**Fig. 3:**
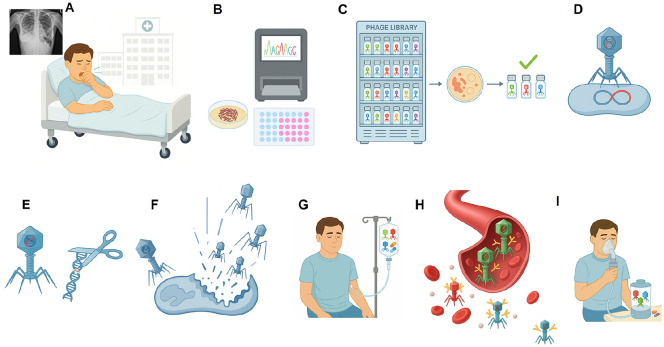
Workflow for personalized phage therapy targeting clinical *M. abscessus* infections. (**A**) A patient presenting with pulmonary symptoms is admitted for clinical evaluation. Chest radiography and other diagnostic assessments reveal pulmonary abnormalities, prompting collection of respiratory specimens for microbiological analysis. (**B**) The clinical isolate obtained from patient specimens was identified as *M. abscessus*, and antibiotic susceptibility testing revealed resistance to multiple therapeutic agents, leading to the consideration of phage therapy as an alternative treatment approach. (**C**) A diverse mycobacteriophage library is screened against the clinical isolate to identify phages exhibiting lytic activity. (**D**) Most *M. abscessus* phages are temperate. (**E**) These temperate phages are genetically engineered to delete the repressor and integrase genes, thereby converting them into lytic phages. (**F**) Engineered lytic phages are combined with naturally lytic phages (such as Muddy) to formulate a patient-specific phage cocktail. (**G**) The phage cocktail is administered intravenously in combination with conventional antibiotics. (**H**) During intravenous therapy, neutralizing antibodies against therapeutic phages may emerge, reducing their systemic bioavailability. (**I**) To overcome antibody-mediated neutralization, the delivery route is switched to aerosolized administration via nebulization, enabling direct pulmonary delivery while minimizing systemic immune inactivation. This illustration was created with an AI-based illustration tool.

To overcome serum neutralization and improve phage delivery to the site of infection in an immunocompetent 81-year-old man whose prior intravenous phage therapy lost sustained efficacy due to strong antibody-mediated neutralization, nebulized administration of the same phage cocktail was initiated. The same triple-phage cocktail (Muddy, BPsΔ33HTH_HRM10, and ZoeJΔ45) was subsequently administered via aerosolization. The nebulized phages were delivered twice daily at a dose of approximately 1 × 10^9^ PFU total in 3 ml of 0.9% saline in combination with ongoing multidrug antibiotic therapy. Nebulized delivery was well-tolerated, with no significant laboratory or imaging abnormalities and only a brief, self-limited episode of hemoptysis unrelated to phage administration (Fig. 3).

Clinical and microbiological monitoring revealed encouraging early responses. Within days of initiating inhaled phage therapy, the patient exhibited improved lung function and decreased sputum production. During the subsequent 3-4 months, his body weight increased by approximately 8.7% (a gain of 4.3 kg from a baseline of 49.7 kg), in parallel with a reduction in sputum bacterial burden, whereas his serum C-reactive protein (CRP) levels declined from 69.3 to 23.9 mg/l, reflecting attenuation of systemic inflammation and aligning with a period of genuine clinical improvement. However, these improvements were not sustained. After approximately three months, the gains in weight, CRP levels, and bacterial counts began to wane and had largely diminished by month 4, paralleling the waning therapeutic effect. Switching from a jet compressor to a mesh-type nebulizer did not improve outcomes. Importantly, longitudinal bacterial isolates remained susceptible to all three phages, excluding the emergence of phage resistance as a cause of failure.

Immunological assessments revealed only weak sputum IgA responses and relatively low levels of local phage neutralization, in clear contrast to the strong systemic IgG- and IgM-mediated neutralization observed during prior intravenous therapy. These findings indicate that aerosolized delivery elicited moderate mucosal antibody activity, permitting phages to persist longer within the respiratory tract. Although mild neutralization increased at later stages, this alone could not explain the transient clinical benefits, suggesting that additional factors, such as limited phage penetration into diseased lung tissue or heterogeneous infection sites, may have also restricted the durability of responsiveness [27].

Taken together, this case demonstrates that aerosolized phage delivery can extend the duration of clinical efficacy in immunocompetent hosts compared with the effects of intravenous therapy while avoiding strong systemic antibody responses. Nonetheless, the ameliorations were transient, highlighting that both the delivery route and host–pathogen interactions shape therapeutic outcomes. Further studies are needed to optimize inhalation strategies, identify eligible patients, and clarify the mechanisms limiting the durability of response.

### Compassionate Use of Bacteriophages for Drug-Resistant *Mycobacterium* Infections

Clinical phage therapy has recently emerged as a compassionate treatment option for NTM infections, particularly *M. abscessus* infections, that are refractory to conventional antibiotics. Building on early individual case reports, a landmark study systematically evaluated personalized phage therapy in 20 patients with refractory NTM infections, most of whom had MABC infections. The majority of these patients had CF, immunodeficiencies, or a history of organ transplantation—populations at the highest risk of progressive and drug-refractory disease.

Phage regimens were individualized by screening each clinical isolate against an extensive panel of naturally isolated or engineered lytic phages. Phages were primarily administered intravenously at approximately 1 × 10^9^ PFUs twice daily, although aerosolized, topical, and bronchoscopic formulations were also used when appropriate. Treatment durations ranged from several weeks to more than a year, with 11 patients receiving therapy for over six months. Favorable clinical or microbiological responses were observed in 11 of 20 patients, including marked improvements in lung function, radiographic findings, and symptom control. For instance, one lung transplant recipient with disseminated *M. abscessus* infection achieved substantial clinical resolution after more than a year of intravenous therapy, while another patient who received combined intravenous and bronchoscopic delivery of Muddy and a BPs derivative achieved sputum culture conversion and pulmonary function recovery. Beyond MABC, phage therapy also proved effective in isolated cases of disseminated *M. chelonae* and *M. avium* complex infection, underscoring its broader therapeutic potential.

Despite these encouraging outcomes, approximately 40% of patients experienced only transient or no clinical benefit. A major challenge was the emergence of neutralizing anti-phage antibodies, particularly in those undergoing prolonged intravenous therapy. Neutralization was confirmed in at least eight patients, and in several cases its onset temporally coincided with clinical deterioration, suggesting a causal link. Notably, nebulized phage delivery appeared to attenuate systemic immunogenicity and was adopted as a secondary strategy in select patients; however, the durability of its therapeutic benefit remained limited. Treatment duration typically ranged from 4 to 9 months for intravenous therapy, with several patients subsequently receiving an additional 6–9 months of nebulized administration, for a total treatment period of up to 15 months [2, 28]. Importantly, no phage resistance emerged in any follow-up isolates, even after extended monophage therapy, which is consistent with *in vitro* findings that rough morphotype *M. abscessus* strains are stably phage-susceptible. In contrast, smooth morphotype isolates remained uniformly resistant to available phages, representing a major barrier to broader clinical implementation [2, 28].

Analysis of the 20 reported cases revealed additional patterns with direct implications for future therapeutic strategies. Therapeutic outcomes varied considerably even among CF patients, indicating that host comorbidity alone is insufficient to predict treatment response and underscoring the need for individualized *in vitro* phage susceptibility testing before therapy initiation. Furthermore, while Muddy was administered to more than half of the patients, outcomes ranged from complete infection resolution to inconclusive, suggesting that Muddy alone is insufficient for reliable efficacy. In contrast, the engineered derivatives BPsΔ33HTH_HRM^GD03^ and D29_HRM^GD40^ were consistently associated with improved responses, especially when incorporated into multi-phage cocktails. Notably, combinations containing BPsΔ33HTH_HRM^GD03^ achieved infection resolution in several patients, supporting the rationale that rationally designed phage cocktails, rather than monotherapy, are critical for maximizing efficacy and minimizing the emergence of resistance (Table 1). Collectively, these observations suggest that the success of phage therapy for *M. abscessus* depends on three interdependent factors:

(i) Precise phage–host matching guided by *in vitro* susceptibility screening,

(ii) Genetic conversion of temperate phages (*e.g.*, BPs and ZoeJ) into obligately lytic forms via deletion of repressor and integrase genes, while naturally lytic phages such as Muddy required no modification, and

(iii) Strategic combination of multiple active phages, ideally delivered by both systemic and localized routes to balance efficacy and immunogenicity.

The consistent association of BPsΔ33HTH_HRM^GD03^, D29_HRM^GD40^, and Muddy with favorable clinical responses positions these phages as promising backbone components for next-generation therapeutic cocktails. Moving forward, controlled clinical trials integrating phage pharmacokinetics, host immune dynamics, and bacterial population structure will be essential to refine dosing strategies and achieve durable clearance of *M. abscessus* infections. Collectively, these findings support that compassionate-use phage therapy is safe, feasible, and capable of producing meaningful clinical and microbiological responses in patients with otherwise untreatable NTM infections, while also highlighting key barriers—namely, the limited repertoire of therapeutic phages, the refractoriness of smooth morphotypes, and host immune neutralization—that must be overcome through continued phage engineering and delivery innovation.

A 2022 case study described the successful use of bacteriophages to treat a disseminated cutaneous infection caused by *M. chelonae* in an immunocompromised adult undergoing hematopoietic stem cell transplantation (HSCT) for myelodysplastic syndrome [29]. *M. chelonae* is a rapidly growing NTM with a close phylogenetic relationship with *M. abscessus*, with both species belonging to the MABC and sharing high genomic similarity [30]. *M. chelonae* is known to cause disseminated cutaneous infection in immunocompromised individuals or localized cutaneous disease in immunocompetent patients [31]. In the 2022 case, the infection, which manifested as multiple non-healing cutaneous nodules on the limbs, had become refractory to long-term combination antibiotic therapy. Because of the patient’s poor clinical response and high risk of progression, the team pursued phage therapy under compassionate use. The *M. chelonae* clinical isolate was subjected to *in vitro* screening against an extensive phage library, and Muddy was the only phage to display potent bactericidal activity. The phage was administered intravenously over several weeks alongside concurrent antibiotics. Remarkably, the patient experienced significant regression of skin nodules within weeks of initiating phage therapy without adverse events or evidence of phage resistance. Notably, anti-phage neutralizing antibodies were not detected during the treatment course, likely because of the patient’s immunosuppressed status post-HSCT.

This case extended the clinical applicability of mycobacteriophage therapy to non-*abscessus* *M. chelonae* infections and supported the feasibility of intravenous phage administration in immunocompromised hosts. It also underscored the potential utility of monophage regimens when only one effective candidate can be isolated from existing phage libraries [29].

### Toward Controlled Clinical Trials: Phage Therapy Study Designs for NTM Pulmonary Disease

Compassionate use applications of bacteriophage therapy in patients with multidrug-resistant *M. abscessus* infections have provided important proofs of concept, but their utility was limited by heterogeneity in treatment regimens, patient populations, and outcome measures. Some patients exhibited microbiological clearance or clinical stabilization, whereas others generated neutralizing antibodies or failed to respond despite receiving phages with activity against their isolates. These experiences underscored the urgent need for standardized clinical protocols to assess safety, efficacy, and mechanisms of response or failure.

The ongoing POSTSTAMP trial (NCT06262282) represents the first prospective, multisite study designed to systematically evaluate mycobacteriophage therapy in patients with CF and refractory *M. abscessus* pulmonary disease [32, 33]. Approximately 10 participants are planned and are being prospectively assessed under FDA IND approval for compassionate use. Eligibility requires persistent infection despite ≥12 months of guideline-based therapy (GBT), ≥80% sputum culture positivity for *M. abscessus* during the prior year, and the ability to produce sputum for longitudinal monitoring. At enrollment, clinical isolates are screened for susceptibility to lytic phages. Matched patients are intravenously receiving one or two phages at 1 × 10^9^ PFUs twice daily for 52 weeks in combination with ongoing GBT. Patients without a phage match are being followed as a comparator group.

The trial incorporates standardized clinical and microbiological endpoints, including sputum culture conversion, the frequency of *M. abscessus* detection, lung function (forced expiratory volume in 1 s), body mass index, pulmonary exacerbations, and patient-reported quality of life using the CFQ-R instrument. Importantly, outcomes are being assessed prospectively using both individual-level comparisons (pre- vs. post-treatment culture positivity) and mixed-effects models across participants. Beyond conventional endpoints, the study also emphasizes biomarker discovery, as serial samples of blood, sputum, urine, saliva, and breath are being banked to analyze culture-independent markers such as cell-free DNA, lipoarabinomannan, volatile metabolites, and host immunoglobulin responses. This integrated approach aims to both quantify treatment efficacy and identify predictors of response and mechanisms of treatment failure, including phage neutralization or bacterial adaptation.

By standardizing the treatment duration (52 weeks), dosing, and endpoints, POSTSTAMP addresses the variability of prior case reports and more objectively evaluates phage therapy. The inclusion of a comparator cohort strengthens interpretation, whereas long-term follow-up (up to 2 years) will permit assessments of both immediate and durable outcomes. Although small in scale, this trial represents a critical first step toward defining efficacy benchmarks, guiding regulatory pathways, and informing the design of larger controlled studies.

In summary, POSTSTAMP operationalizes lessons from compassionate use experiences into a structured clinical framework. Its prospective design, rigorous monitoring, and emphasis on mechanistic correlates will provide essential data to determine whether phage therapy can move from individualized interventions to reproducible treatment strategies for refractory *M. abscessus* pulmonary disease. As of the most recent update (May 2025), the trial remains active, and no preliminary or outcome data have yet been released [32].

### Next-Generation Delivery Strategies: LysB Enzymes and Liposomal Encapsulation for Intracellular Phage Therapy

The therapeutic use of bacteriophages against *M. abscessus* infection faces a critical limitation, namely the pathogen’s predominant intracellular localization within macrophages and epithelial cells. To address this, recent research has shifted toward next-generation delivery strategies that enhance intracellular access [34, 35], circumvent immune clearance [34, 36, 37], and bypass the requirement for whole phage replication cycles [35, 38, 39]. Among these, direct administration of phage-derived lytic enzymes and encapsulation of phages in liposomal carriers have emerged as key approaches.

### Mycobacteriophage-Derived LysB

Mycobacteriophage LysB is a lytic enzyme that cleaves the ester linkage between mycolic acids and the arabinogalactan layer of the mycobacterial outer membrane. Unlike intact phage particles, which require adsorption and replication, LysB induces direct enzymatic degradation of the bacterial cell envelope. Hurst-Hess *et al*. demonstrated that aerosolized intrapulmonary delivery of purified D29 LysB at 40 μM (50 μl daily for 6 days) in SCID mice significantly reduced the *M. abscessus* lung burden. During the 9-day experimental period, untreated mice showed a natural ≈10-fold decline in bacterial load, whereas LysB administration produced an additional ≈20-fold reduction relative to controls [35]. LysB treatment improved pulmonary pathology and decreased inflammatory infiltration, but the study assessed safety only by monitoring body weight (<10 % loss) and clinical activity rather than detailed immune parameters [35].

### Liposome-Encapsulated Phage Therapy

Although certain mycobacteriophages can cross epithelial barriers and be internalized by mammalian cells, their intracellular activity remains inefficient and transient [40]. Traditional phage therapy, relying primarily on extracellular lysis and passive diffusion, therefore cannot fully eradicate intracellular bacterial reservoirs.

Liposomal encapsulation provides both mechanistic and immunological advantages that justify its application to intracellular mycobacterial infections. Mechanistically, liposomes facilitate phage uptake by macrophages through endocytic and phagocytic pathways, as demonstrated by Nieth *et al*. [41], who showed that liposome-associated mycobacteriophages are internalized more efficiently than free virions and accumulate within endosomal compartments that intersect with mycobacterial phagosomes. This spatial proximity between phage-containing endosomes and phagolysosomes enhances opportunities for phage–bacterium interaction, thereby improving intracellular killing efficiency. Consistent with this mechanism, Vladimirsky *et al*. demonstrated that liposome-encapsulated mycobacteriophage D29 exhibited nearly tenfold higher bactericidal activity against intracellular *M. tuberculosis* in RAW 264.7 macrophages compared with free phages, confirming the benefit of lipid-mediated delivery in overcoming endosomal barriers [42]. Similarly, Silva *et al*. showed that nanoliposome-loaded D29 achieved over 90% reduction in intracellular *M. tuberculosis* burden without cytotoxicity and remained active against both replicating and dormant bacilli [43]. These studies collectively support that liposomal encapsulation not only enables phages to reach otherwise inaccessible intracellular niches but also shields them from serum or mucosal neutralization, ensuring sustained bactericidal efficacy. In the context of *M. abscessus*, Schmalstig *et al*. reported that only a subset of phages can naturally penetrate macrophages and achieve partial intracellular killing, suggesting that passive transcytosis and epithelial uptake alone are insufficient for meaningful therapeutic outcomes [40]. Thus, liposomal encapsulation serves as both a protective vehicle and a controlled-release platform, enhancing phage persistence and enabling targeted delivery within macrophage phagosomes.

From an immunological standpoint, increased macrophage internalization of liposome-encapsulated phages may also influence host immune dynamics. Van Belleghem *et al*. demonstrated that phages interact directly with innate immune cells and can induce anti-inflammatory cytokines such as IL-1 receptor antagonist (IL1RN) and IL-10, while suppressing excessive TNF-α and IL-6 production. These interactions suggest that enhanced uptake of liposomal phages could facilitate localized immunomodulation within infected tissues while minimizing systemic inflammatory responses. At the adaptive level, internalized or degraded phage capsid proteins may be processed by antigen-presenting cells, potentially stimulating mild and transient humoral responses-a reflection of the inherently low immunogenicity of purified phage particles [44].

Nevertheless, trafficking of liposome-encapsulated phages to lysosomes represents a double-edged process. While lysosomal degradation can restrict phage survival, it may simultaneously promote antigen presentation and macrophage activation, potentially enhancing immune priming against intracellular mycobacteria. Optimizing liposomal composition, charge, and size to balance phage protection, endosomal escape, and immune compatibility therefore remains a critical step toward maximizing intracellular delivery and therapeutic efficacy.

**Fig. 4:**
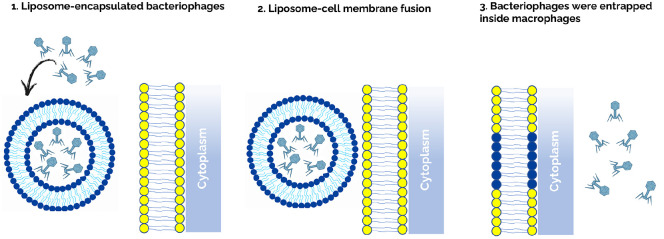
Liposome-mediated intracellular delivery of bacteriophages into macrophages. (**A**) Bacteriophages are encapsulated within phospholipid bilayers to generate liposomal formulations that mimic host-derived vesicles. This lipid encapsulation enhances phage stability and protects virions from antibody- or serum-mediated neutralization. (**B**) Following administration, the liposomal surface interacts with macrophage membranes through recognition of phospholipid and charge features, promoting liposome–cell membrane fusion and phagocytic uptake. (**C**) Once internalized, the liposomal membrane fuses with the phagosomal membrane, enabling the release of phages directly into the intracellular compartment where *M. abscessus* resides. This targeted delivery facilitates efficient intracellular access and sustained antibacterial activity, overcoming one of the major barriers to effective phage therapy against intracellular mycobacteria. This illustration was created with an AI-based illustration tool.

In summary, liposome encapsulation constitutes a rational and experimentally validated approach for improving the stability, intracellular access, and immunological tolerance of therapeutic phages. Integration of liposomal or nanocarrier-based formulations with engineered mycobacteriophages represents a logical progression toward overcoming one of the key barriers in phage therapy—achieving efficient and sustained bactericidal activity against macrophage-resident *M. abscessus* (Fig. 4).

### Clinical Lmplications and Future Outlook

Both LysB-based lytic enzyme therapy and liposome-encapsulated phage delivery offer mechanistically distinct but complementary solutions to the challenge of treating intracellular mycobacterial infections. The former approach provides a rapid bacteriolytic effect without the need for phage replication, whereas the latter ensures the safe delivery of intact phages across cellular membranes and immunological checkpoints. As these technologies advance toward clinical translation, their integration with conventional antibiotics, immunomodulatory agents, or inhalable delivery platforms could enhance the precision and efficacy of future anti-*M. abscessus* treatment protocols.

### Synergistic Interplay between Bacteriophages and Antibiotics in the Treatment of *M. abscessus*

The multidrug-resistant nature of *M. abscessus* has prompted growing interest in bacteriophage therapy as an adjunct to conventional antibiotics. Rather than functioning as standalone agents, phages act synergistically with select antibiotics, offering enhanced bacterial clearance both *in vitro* and *in vivo*. This combination strategy is particularly relevant in the context of chronic, refractory infections, which frequently fail to respond to monotherapies.

Johansen *et al*. investigated this concept using the lytic phage Muddy in combination with several antibiotics against *M. abscessus* subsp. *massiliense* GD01, a strain isolated from a patient with CF having disseminated disease. *In vitro* assays demonstrated significant synergy between the phage and antibiotics, such as rifabutin, imipenem, and tigecycline, demonstrated significant synergy[45]. This synergy translated into improved survival and reduced bacterial burden in a CFTR-deficient zebrafish model, and therapeutic efficacy was abolished in macrophage-depleted larvae [45].

Further supporting this approach, Cristinziano and colleagues reported a lung-transplant recipient with multidrug-resistant *M. abscessus* sternal wound infection successfully treated with two therapeutic phages—Muddy and a host-methylated (“epigenetically compatible”) BPsΔ33HTH_HRM10^pMC09^—administered intravenously in combination with dual β-lactam therapy (meropenem plus ceftazidime-avibactam). The treatment was well tolerated and led to marked clinical and radiographic improvement with no evidence of progressive disease during therapy[46].

Building on these findings, Gorzynski and colleagues investigated the molecular mechanisms underlying phage–antibiotic synergy in *M. abscessus*. Their study revealed that phage infection induces bacterial envelope remodeling and stress responses that sensitize the pathogen to concurrent antibiotic exposure. Transcriptomic and proteomic analyses showed downregulation of genes associated with efflux pump function and DNA repair, together with alterations in lipid metabolism that compromise cell wall integrity. These intracellular perturbations increase antibiotic permeability and potentiate bactericidal activity. Conversely, antibiotics that disrupt peptidoglycan cross-linking or interfere with protein synthesis facilitate phage adsorption and replication, establishing a bidirectional enhancement loop between the two modalities. Collectively, these results provide mechanistic support for integrating bacteriophage therapy with antibiotics to achieve improved clearance of *M. abscessus*, particularly in biofilm-rich and intracellular environments [47].

**Fig. 5:**
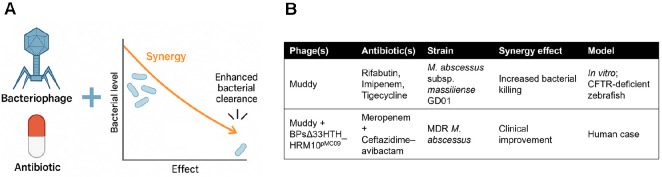
Synergistic activity of combined phage–antibiotic therapy. (**A**) Schematic depiction showing that concurrent administration of bacteriophages and antibiotics yields superior bacterial reduction compared with monotherapy, reflecting a synergistic therapeutic interaction. (**B**) Summary of phage–antibiotic combinations evaluated across distinct *M. abscessus* strains and experimental settings. The compiled examples demonstrate conditions under which enhanced bactericidal activity has been observed, integrating both *in vitro* assessments and clinical observations. Together, these data illustrate that synergy between phages and antibiotics can occur across multiple therapeutic contexts and pathogen backgrounds, supporting the rationale for combination approaches. This illustration was created with an AI-based illustration tool.

Overall, growing evidence supports the integration of phage therapy into standard antimicrobial regimens for *M. abscessus*, particularly when guided by host–phage compatibility, strain-specific resistance patterns, and phage–drug interaction profiling. This synergy offers new hope for otherwise untreatable infections and provides a rational framework for future clinical trial design (Fig. 5).

## Discussion

Bacteriophage therapy for MABC infections has progressed from theoretical promise to clinical proof of concept through a growing body of compassionate use cases. Early individual cases and the larger 20-patient cohort demonstrated that phage therapy is generally safe, well-tolerated, and capable of producing meaningful microbiological and clinical benefits, including improvements in lung function, radiographic stability, and symptom control [19, 22, 23, 26-28]. These findings challenged long-standing doubts about whether phages could access lipid-rich, intracellularly localized mycobacteria [19, 20]. However, variability in treatment regimens, patient populations, delivery routes, and outcome measures, coupled with the frequent emergence of neutralizing antibodies, particularly during intravenous administration, has highlighted the heterogeneity and limitations of the current evidence base [26-28]. In essence, existing studies confirm that phage therapy is effective, but the conditions under which it works consistently, optimal patient population, and duration of efficacy have not yet been established. Several phages have emerged as the principal candidates in compassionate-use treatment of MABC infections, most notably D29, Muddy, BPs, and ZoeJ [19, 28, 29]. These phages share key therapeutic features, including obligately lytic biology, rapid adsorption, and short replication cycles. Muddy is a naturally isolated phage, whereas the engineered phages used clinically lack integrases, repressors, and other lysogeny-associated genes, confirming a strictly lytic lifestyle and reducing concerns regarding genomic integration [2, 8, 20]. Engineered variants such as the lytic ZoeJ mutant further demonstrate the feasibility of optimizing phage genomes for enhanced safety and performance [19]. The infection kinetics and genomic simplicity of these phages underpin their repeated use in clinical *M. abscessus* management and provide a foundation for the development of next-generation therapeutic phages. Although clinical cases have not yet documented the emergence of phage resistance, laboratory studies indicate that *Mycobacterium* spp. possess several potential resistance pathways. Recent work has shown that *M. abscessus* can alter surface-exposed GPL(glycopeptidolipid) and other cell envelope components, reducing phage adsorption efficiency and conferring morphotype-dependent resistance [48]. Additional studies have identified mutations in genes involved in cell wall biosynthesis, stress responses, and phage receptor pathways, which can collectively restrict phage binding or impede DNA injection. Phenotypic adaptations-including smooth-to-rough switching, altered GPL expression, and changes in colony morphology-also modulate susceptibility and may influence resistance trajectories under therapeutic pressure. While such mechanisms highlight the genetic and structural flexibility of *Mycobacterium*, the absence of resistance in clinical reports likely reflects careful pre-treatment susceptibility screening, combination therapy with antibiotics, and the limited replication capacity of phages in heavily diseased tissue. Together, these findings underscore the importance of serial susceptibility testing and genomic monitoring during treatment to detect emerging resistance early and guide phage selection.

The most prominent barriers to efficacy are immunological neutralization and morphotype-dependent susceptibility. Intravenous administration, although logical for disseminated infections, frequently elicited robust systemic IgG- and IgM-mediated neutralizing responses that correlated with clinical relapse [26]. Inhaled delivery reduced systemic immunogenicity and allowed phages to persist longer in the respiratory tract, but the benefits remained transient, likely because of incomplete tissue penetration or a heterogeneous infection site distribution [27]. A growing body of work now suggests that these biological barriers can be strategically overcome through rational engineering and formulation approaches. Immune neutralization, in particular, can be mitigated through the use of long-circulating capsid variants, pulsed or alternating phage dosing regimens, and biodegradable encapsulation systems designed to shield phages from antibody recognition. Early foundational studies showing that long-circulating mutants can evade reticuloendothelial clearance and markedly extend *in vivo* persistence [49], together with more recent evidence that PLGA/alginate composite carriers enable sustained release while protecting phages from neutralizing antibodies for prolonged periods [50], collectively underscore an emerging principle: effective, durable phage therapy will require immune-informed delivery system design. Such strategies will be essential for maintaining therapeutic phage titers during prolonged treatment courses.

With regard to bacterial cells, rough MABC morphotypes are generally phage-susceptible, whereas smooth morphotypes remain uniformly resistant to currently available phages, restricting the breadth of clinical applicability [28, 30]. These barriers underscore the need for future trials to stratify patients based on their immune status (immunocompromised vs. immunocompetent), delivery route (intravenous vs. inhaled or combined), and morphotype (rough vs. smooth) to enable more precise interpretation of therapeutic outcomes. Historically, morphotype-dependent susceptibility has been interpreted through the long-standing assumption that smooth (S-type) *M. abscessus* strains are intrinsically resistant to phage infection because their GPL-rich outer layer masks potential phage receptors. Indeed, phages capable of infecting S-type strains had not been identified from natural environmental isolates, reinforcing the notion that the presence or absence of GPL constitutes a decisive barrier to phage entry [48]. However, recent studies demonstrate that this dichotomy is not absolute. Several newly characterized mycobacteriophages have shown efficient adsorption and productive killing in S-type clinical isolates, indicating that certain receptor-binding proteins can partially or fully bypass the GPL barrier [47, 51]. These findings suggest that smooth morphotypes cannot be categorically regarded as “phage-resistant,” and that susceptibility is more phage-specific and structurally nuanced than previously assumed. This emerging evidence supports the feasibility of expanding phage libraries with smooth-active isolates, engineering receptor-binding proteins to overcome GPL masking, and developing dedicated smooth-targeting phages. Given that S-type variants frequently arise early during infection and often coexist with rough subpopulations, incorporating smooth-active phages into therapeutic design will be essential to achieving consistent and durable clinical outcomes [52].

Delivery and formulation comprise another axis of challenge. Intravenous therapy remains relevant for disseminated disease, but its efficacy is compromised by systemic immunogenicity. Inhalation provides more direct targeting but faces limitations regarding tissue penetration and device-dependent viability [27]. Emerging next-generation strategies, such as phage-derived lytic enzymes (*e.g.*, LysB) [35] and liposome-encapsulated phages [34, 41], aim to bypass these barriers. LysB, a recombinant D29-derived esterase, achieved approximately a 20-fold reduction in *M. abscessus* lung burden in SCID mice when administered at 40 μM via aerosol, confirming potent replication-independent lysis, whereas liposomal encapsulation of TM4 phages enhanced macrophage internalization and protected virions from antibody-mediated neutralization [34, 35, 41]. Clinical translation of these approaches will depend on GMP-grade production, delivery standardization, and development of pharmacokinetic/pharmacodynamic markers to verify target site exposure [21].

Another critical insight is the synergistic potential of phage–antibiotic combinations. Pre-clinical studies demonstrated that phages can act synergistically with antibiotics, such as rifabutin, imipenem, or tigecycline, to enhance bacterial clearance [45], whereas clinical findings confirmed durable responses when phages were combined with multidrug regimens [19, 28, 46]. Mechanistic studies by Gorzynski *et al*. suggest that antibiotics can increase phage susceptibility by disrupting cell wall integrity or metabolic homeostasis, whereas phages can augment antibiotic efficacy by degrading biofilms or impairing efflux systems [47]. This reciprocal interaction reframes phages as complementary agents within multidrug treatment frameworks. Moreover, although phage–antibiotic synergy has been extensively documented across diverse bacterial pathogens-including *Pseudomonas aeruginosa*, *Acinetobacter baumannii*, *Staphylococcus aureus*, and *Escherichia coli*-comparable studies in *Mycobacterium* spp. remain surprisingly scarce [53]. The robust synergistic effects observed in these organisms, such as antibiotic-induced filamentation facilitating enhanced phage predation or phage-mediated restoration of antibiotic susceptibility, provide a strong conceptual basis for combination therapy [33, 53-55]. Importantly, clinical experience with *M. abscessus*—including all compassionate-use cases—has relied on concurrent antibiotic therapy rather than phage monotherapy. This indicates that combination therapy is already the de facto clinical standard, yet the field lacks systematic evidence identifying which antibiotic classes, dosing strategies, or pharmacodynamic interactions most effectively potentiate mycobacteriophage activity. Dedicated synergy studies tailored to mycobacterial physiology are therefore urgently needed to establish rational, evidence-based combination regimens and to move beyond empiric continuation of background antibiotics.

POSTSTAMP represents an important step toward addressing the heterogeneity of compassionate use cases [28]. By standardizing the phage dose (1 × 10^9^ PFUs twice daily), treatment duration (52 weeks), and study endpoints (culture conversion, lung function, quality of life) while incorporating biomarker discovery and comparator cohorts, POSTSTAMP establishes a structured platform for systematic evaluation. Its integration of culture-independent biomarkers, including cell-free DNA, lipoarabinomannan, volatile metabolites, and host immune responses, will provide valuable mechanistic insights into both treatment success and failure. Although modest in scale, POSTSTAMP exemplifies how lessons from compassionate use can be operationalized into reproducible trial design.

Several priorities should be evident for future development. First, strategies to mitigate immune neutralization, such as phage alternation, encapsulation, pulsed dosing, and selective immunomodulator use, must be systematically evaluated [26-28, 34, 41]. Second, the therapeutic repertoire must be expanded to overcome smooth morphotype resistance through novel phage isolation, host range engineering, and synthetic biology approaches [21, 28, 46]. Third, regulatory science must address GMP consistency, endotoxin management, and inhaled delivery standards to enable scalable clinical application [21]. Finally, given the challenges of randomized controlled trials in this rare and heterogeneous patient population, adaptive trial designs, platform studies, and registry-based approaches may be needed to accelerate evidence generation [15, 16].

Current evidence indicates that bacteriophage therapy, when tailored to the host immune status, morphotype, and delivery route, can provide meaningful clinical benefits in otherwise untreatable *M. abscessus* infections. However, its reproducibility and durability remain constrained by immunological and biological barriers. Moving forward, bacteriophage therapy appears to have entered a stage of cautious optimism rather than experimental novelty. Compassionate use cases and preclinical studies have confirmed its safety and therapeutic promise, yet the field remains fragmented by immunological, microbiological, and regulatory challenges.

Future progress will depend on achieving four converging goals. First, defining the *in vivo* pharmacokinetic and pharmacodynamic behavior of phages and lytic enzymes will be essential to establish rational, evidence-based dosing strategies. Second, elucidating the immunological interplay between phages and the human host-particularly the mechanisms of antibody neutralization and mucosal immunity-will inform the development of optimized delivery systems. Third, expanding and engineering phage libraries with broader host ranges will be critical to overcome smooth morphotype resistance and mixed-strain infections. Finally, integrating phage therapy within multidrug regimens through well-controlled, adaptive clinical trials will be necessary to validate reproducibility and durability of therapeutic benefit.

Addressing these challenges will determine whether bacteriophages can evolve from a compassionate-use intervention into a standardized, mechanistically guided treatment platform for multidrug-resistant nontuberculous mycobacterial disease.

## Figures and Tables

**Table 1:** Clinical profiles and outcomes of 20 patients with refractory NTM infections treated with compassionate-use phage therapy.